# Myocardial salvage in acute myocardial infarction assessed by magnetic resonance imaging – influences by the antioxidative agent N-acetylcystein

**DOI:** 10.1186/1532-429X-11-S1-O27

**Published:** 2009-01-28

**Authors:** Holger Thiele, Ingo Eitel, Lysann Hildebrand, Carmen Schirdewahn, Volker Adams, Georg Fürnau, Matthias Gutberlet, Gerhard Schuler

**Affiliations:** 1 University of Leipzig – Heart Center, Leipzig, Germany; 2 Internal Medicine – Cardiology, Leipzig, Germany

**Keywords:** Infarct Size, Surrogate Endpoint, Microvascular Obstruction, Delayed Enhancement, Myocardial Salvage

## Introduction

Myocardial salvage can be assessed retrospectively by T2-weighted and delayed enhancement images as shown in animal studies [1]. Currently there are only limited data in humans with acquisition of T2-weighted images not covering the full ventricle [2]. Animal trials showed that the antioxidant effects of N-Acetylcystein (N-ACC) reduce reperfusion injury and N-ACC might prevent the occurrence of contrast-induced nephropathy (CIN) in patients undergoing primary PCI.

## Purpose

Aim of this trial was to establish myocardial salvage imaging by MR as a surrogate endpoint in a randomized single-blinded trial and to show that high-dose N-ACC reduces reperfusion injury by its antioxidative properties. Furthermore, N-ACC might reduce the incidence of contrast-induced nephropathy (CIN).

## Methods

Two hundred-fifty-one patients undergoing primary PCI were randomized to either high-dose N-ACC (2 × 1200 mg/d for 48 hours) or placebo plus optimal hydratation. The two primary endpoints were: 1) occurrence of CIN defined as an increase in the serum creatinine concentration of >25% from the baseline value within 72 h; 2) Myocardial salvage measured by T2-weighted STIR-images (covering the left ventricle from base to apex) and delayed enhancement MRI at day 2–4 after primary PCI. Myocardial salvage index was calculated as area at risk-infarct size/area at risk.

Secondary endpoints were infarct size and microvascular obstruction, ST-resolution at 90 minutes and occurrence of MACE at 30 day follow-up.

## Results

Due to contraindications MRI could not be performed in 31 patients. All images were assessable for the calculation of the myocardial salvage index. The area at risk was 47.3% of the left ventricular mass (IQR 33.9; 58.8) in the N-ACC group versus 42.7% (IQR 33.7; 53.0; p = 0.39) in the placebo group. The primary endpoint reperfusion injury measured by myocardial salvage index was not different between both treatment groups (57.7; IQR 39.2; 78.0 versus 61.1; IQR 40.6; 77.7; p = 0.32). In addition, no differences in infarct size (18.3%; IQR 9.3; 26.4 versus 14.9%; IQR 8.0; 26.4; p = 0.48) and microvascular obstruction (0.7%; IQR 0.2; 1.5 versus 0.6%; IQR 0.0; 1.2; p = 0.25) as well as in ST-segment resolution were observed. The median volume of an iso-osmolar contrast agent during PCI was 190 ml (IQR 130, 250 ml) in the N-ACC and 180 (IQR 143; 228 ml) in the placebo group (p = 0.67). The primary endpoint CIN occurred in 14% in the N-ACC group and in 22% in the placebo group (p = 0.12).

## Conclusion

MRI can reliably measure the area at risk and infarct size retrospectively and served as a surrogate endpoint in this randomized clinical trial which showed that high-dose N-ACC does not provide an additional clinical benefit to placebo with respect to prevention of myocardial reperfusion injury and CIN and in patients undergoing primary PCI.
